# Psychopathology, Body Image and Quality of Life in Female Children and Adolescents With Anorexia Nervosa: A Pilot Study on the Acceptability of a Pilates Program

**DOI:** 10.3389/fpsyt.2020.503274

**Published:** 2020-10-30

**Authors:** Sofía M. Martínez-Sánchez, Concha Martínez-García, Tomás E. Martínez-García, Diego Munguía-Izquierdo

**Affiliations:** ^1^Department of Sports and Computer Science, Faculty of Sports Sciences, Section of Physical Education and Sports, Universidad Pablo de Olavide, Seville, Spain; ^2^Department of Social, Evolutionary, and Educational Psychology, Faculty of Psychology, Education and Sports Sciences, University of Huelva, Huelva, Spain; ^3^Department of Internal Medicine, Juan Ramón Jiménez Hospital, Huelva, Spain; ^4^Department of Sports and Computer Science, Faculty of Sports Sciences, Physical Performance Sports Research Center, Section of Physical Education and Sports, Universidad Pablo de Olavide, Seville, Spain; ^5^Biomedical Research Networking Center on Frailty and Healthy Aging, Madrid, Spain

**Keywords:** anorexia nervosa, pilates, psychopathology, body dissatisfaction, quality of life, children, adolescents

## Abstract

**Background:** Anorexia nervosa (AN) is a psychiatric illness that without early effective treatment becomes chronic with high physical, psychological and social morbidity and high mortality. Pilates exercises can improve quality of life and increase body awareness in different clinical and healthy populations. The aim of this pilot study was to examine the acceptability of a Pilates program in a sample of female children and adolescents with AN by evaluating the psychopathological status, alterations in the perception of body image and health-related quality of life after 10 weeks.

**Methods:** A total of 12 female patients (age: 14.6 ± 1.7 years) completed the 10-week Pilates program. Psychopathology (EDI-3), body image disturbance (CDRS) and quality of life (KIDSCREEN-27) were evaluated before and after the intervention. A satisfaction questionnaire was also provided.

**Results:** Regarding psychopathology, although there were standardized reductions in seven parameters of those that form EDI-3, none of them reached significance. In relation to body image, significant, moderately standardized and substantial decreases were observed in the body dissatisfaction (*p* = 0.046, Cohen's *d* = −0.69). There were significant, large standardized and substantial increases in physical well-being (*p* = 0.008, Cohen's *d* = 1.37) and significant, moderately standardized and substantial decreases in autonomy and parent relation (*p* = 0.021, Cohen's *d* = −0.60). Satisfaction data was positive.

**Conclusion:** A Pilates program could help to improve perceived health outcomes by decreasing body dissatisfaction and increasing physical well-being in female children and adolescents with AN, so Pilates seems to be a beneficial complementary treatment in children and adolescents with AN. These findings from our pilot study are encouraging for future research with a substantially larger sample size, representing the first phase of a longer process.

## Introduction

Anorexia nervosa (AN) is a psychiatric illness that includes in its description an intense fear of gaining weight, and a disturbance in the way in which one's body shape is experienced or a persistent lack of recognition of the seriousness of the low body weight (1). The lifetime prevalence of AN in the general population is reported to be ~1% among women and occurs predominantly in adolescence in the peripubertal period (2). Thin ideal internalization, weight-related teasing or general concerns about weight and appearance are some of the triggers for AN in children and adolescents (3). Somatic and mental consequences of the illness at an early age probably have a deleterious effect on later adult life (4). Without early effective treatment, the illness becomes chronic with high physical, psychological and social morbidity and high mortality (2).

Body image disturbance is a robust predictor of AN, illness relapse and often persists in otherwise recovered patients (5, 6). This body image disturbance is associated with body dissatisfaction of the person based on negative thoughts about his or her own body (7). In addition, body image concerns and interoceptive processing also seem to be linked, whereby poor body awareness can aggravate body image disturbances in AN (8). Therefore, treatment and prevention programs should aim to moderate the overvaluation of “thinness” and body dissatisfaction as one of the proximal risk factors (9).

Patients with AN report poorer health-related quality of life (HRQoL) compared to both the general population and other psychiatric/somatic diseases (10). Patients who apparently obtain complete remission will still be affected in HRQoL when compared to a healthy reference group (10). Lower BMI and higher levels of organic or psychiatric comorbidities seem to be associated with a lower HRQoL than age, diagnostic subtype, duration or psychopathology of the illness or current psychiatric treatment (10).

Physical activity has been observed to have broad positive effects on children and adolescents, improving psychological functioning, body acceptance, quality of life, and physical and mental health in general (11–15). Likewise, resistance training, yoga or body awareness therapies have been found to be beneficial in adolescents with AN (16–18). Pilates is a mind-body exercise in which special attention is required to postural control, body movement and breathing (19). It has been evidenced that Pilates exercises can improve quality of life and increase body awareness in different clinical and healthy populations, including children (20–26). Moreover, Pilates can improve perceived body image positively, thereby increasing satisfaction with body self-image (27). The benefits of a Pilates program mentioned above have mainly been aimed at improving the symptomatology of people with other psychopathological diagnoses and the non-clinical population. However, its possible acceptability in children and adolescents with AN have not been studied, when it is known that they share symptoms such as body dissatisfaction and loss of quality of life, among others. Therefore, the aim of this pilot study was to examine the acceptability of a Pilates program in a sample of female children and adolescents with anorexia nervosa by evaluating the psychopathological status, alterations in the perception of body image and health-related quality of life after 10 weeks.

## Methods

### Sample

**Figure 1:**
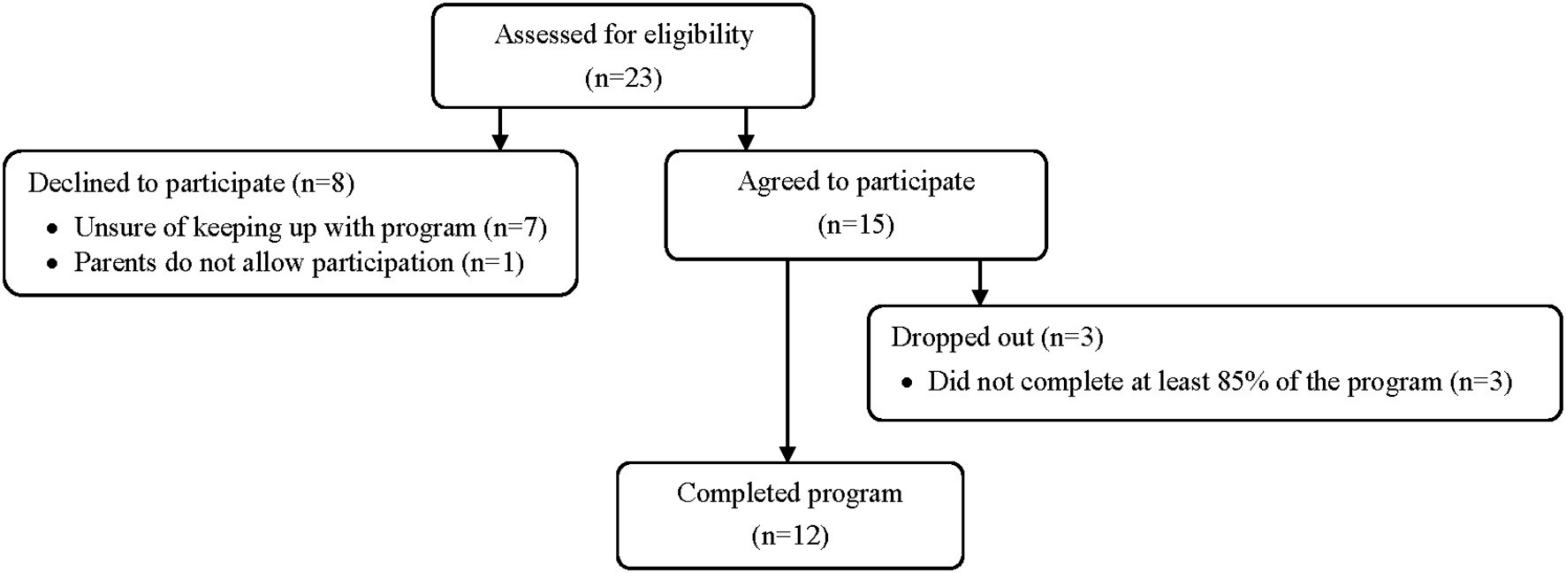
Flow diagram of the study patients.

All female patients between 10 and 17 years old with a clinical AN diagnosis (*n* = 23) were recruited from the Child-Mental Health Unit of the Vázquez Díaz Hospital (Huelva, Spain). The clinical criteria according to the fifth edition of the Diagnostic and Statistical Manual of Mental Disorders (DSM-5) (1) were followed by an experienced psychiatrist. The inclusion criteria were: (1) clinical diagnosis of AN in the aforementioned hospital; (2) aged from 10 to 17 years old; (3) the medical team gave the approval under analytical control and body mass stability; and (4) written informed consent by the patients and their legal guardians. The exclusion criteria were (1) any psychiatric diagnosis (apart from AN) and (2) consumption of narcotic toxins. Fifteen eligible patients for this pilot study agreed to participate and signed written informed consent and their parents (Figure 1). This study was part of a Clinical Research, which was approved by the Research Ethics Committee of the University Hospital Complex of Huelva (PI 005/16), registered in www.clinicaltrials.gov (Identifier: NCT03667183) and the guidelines of the Declaration of Helsinki were followed, last modified in 2013. All patients were assessed by the same researcher to reduce interexaminer error. A code was assigned to each patient to blind the data in the statistical management.

### Pilot Study Procedure

This was a small uncontrolled pilot study. For each patient, a visit was scheduled where anthropometric data and questionnaires were collected (Pre). After the Pilates program, they were asked to complete the questionnaires again (Post). The time between admission to the hospital and the approval of the medical team to enter the Pilates program was 118.7 (± 11.6) days on average. During that time, patients received cognitive behavioral therapy and continued in it throughout the program. All patients were informed about the voluntary character of their participation and were assured that all the data were gathered anonymously.

### Pilates Program

The Pilates program was conducted in group for an hour, three times a week for 10 consecutive weeks by a certified physiotherapist as Pilates Instructor. The exercises were performed on mats and each movement was repeated 10 times. Patients were instructed to perform the movements with control and precision, with attention to their breathing pattern (exhaling during flexion and inhaling during extension, for example) and to core control activation during execution (28). The detailed protocol is explained in Supplementary Material.

### Anthropometry

Standing height was measured to the nearest 0.1 cm following standard procedures using a balance with an incorporated stadiometer (Detecto 439; Detecto, USA), while subjects were standing barefoot. Weight was determined to the nearest 0.05 kg through bioelectrical impedance analysis (InBody 770; Inbody Co., LTD, Seoul, Korea) with the subject in their underwear. Body mass index (BMI) was calculated as weight (kg) divided by height (m) squared.

### Measures

EDI-3 (Eating Disorder Inventory-3) (29) is a self-report questionnaire that assesses the presence of eating disorders (EDs) and analyzes psychological traits and key symptoms considered relevant in the development and maintenance of eating disorders, including AN. It is composed of 91 items with a choice of six answers organized into 12 main scales: three specific scales of EDs and nine general psychological scales that are highly relevant but not specific to EDs. It also provides six composite scores: a specific one of the EDs and five indices of integrative psychological constructs. We used the Spanish version of the EDI-3 (30), which has high levels of internal consistency in all the diagnostic groups (Cronbach's alpha from 0.85 to 0.95). EDI-3 is also one of the most used tools worldwide to monitor the evolution of EDs, and it has been used as an assessment tool in young Spanish (31).

The Contour Drawing Rating Scale (CDRS) (32) is one of the most commonly used, easy-to-administer, and most popular figure drawing scales for the evaluation of body image disturbance (33). This self-administered scale is composed of nine female figures views from the front with relatively fine increasing graduations that extend from the representation of a BMI < 18.5 kg/m^2^ (very low weight) up to a BMI > 30 kg/m^2^ (obesity). The person is asked to indicate which figure represents their current body shape (perceived body shape) and what figure they would like to have (ideal body shape). The discrepancy between these two classifications represents a measure of body dissatisfaction. A score of 0 was interpreted as satisfaction with body image, and a score different than 0 was interpreted as body dissatisfaction (Satisfied = 0; Dissatisfied = 1) (34). The validity and reliability test-retest of the CDRS have been satisfactorily analyzed in female children and adolescents (Cronbach's alpha = 0.77–0.84) (33), and it has been widely used in clinical and healthy populations (35).

To evaluate the HRQoL, the Spanish version of KIDSCREEN-27 was used. KIDSCREEN-27 has been previously used in adolescents with EDs as a measure of quality of life (36). It is an internationally validated instrument, and its Spanish version obtained an acceptable reliability (Cronbach's alpha = 0.78–0.84) (37) and it is composed of five dimensions on the Rasch scale. Raw scores were transformed based on standard algorithms for each dimension and used to compute T-scores, with a mean of 50 and a standard deviation of 10. Higher scores indicate a higher HRQoL. A difference of half an SD (38) or a five-point difference on the dimensions are considered clinically relevant.

We also provided a satisfaction questionnaire about the Pilates program performed at Post-assessment. It included the following 3 questions: “1. Do you think Pilates has improved your posture (walk better, sit better) and flexibility?; 2. Do you think Pilates has helped you into feeling better?; 3. Do you think Pilates has made you have less back pain?” Each question had five possible Likert-scale responses rated from 1 to 5 points: “strongly disagree” (1), “disagree” (2), “neither agree nor disagree” (3), “agree” (4) or “strongly agree” (5).

### Statistical Analysis

All statistical analyses were performed with SPSS 24.0 (IBM Corporation. IBM SPSS Statistics for Windows. Armonk, NY, USA). Significance was set at *p* < 0.05. The Shapiro-Wilk test was used to test for normality of distribution. The Kruskal-Wallis test was used to compare demographic data among the patients who completed the program, those dropped out, and those who declined to participate in the study. The Wilcoxon test was used to evaluate the significant differences between the Pre and Post Pilates program. Data were also assessed for practical/clinical meaningfulness using an approach based on the magnitudes of change. Cohen's d statistic determined the effect size of the standardized differences in the selected variables, and Hopkins' scale (39) and a customized spreadsheet (40) were used to determine the magnitude of the effect size. A practically worthwhile difference was assumed when the difference score was at least 0.2 of the between subject standard deviation. Threshold values for Cohen's effect size were trivial (0.0–0.19), small (0.20–0.59), moderate (0.60–1.19), large (1.20–1.99), and very large (≥2.00). Quantitative chances of positive/trivial/negative difference were assessed qualitatively as follows: <25% unclear, 25–75% possibly, >75% likely, >95% very likely, and >99.5% almost certainly. A substantial difference was set at >75%. The bivariate Spearman's correlation coefficients were used to evaluate the correlations of the difference of each parameter between the three groups of variables analyzed. A value larger than 0.7 in the correlation coefficient was considered a strong correlation (41).

## Results

### Sample Characteristics

Of the 15 patients included in the intervention, three of them were excluded in this pilot study for not completing at least 85% of the intervention. The program was completed by 12 female children and adolescents diagnosed with AN, with an age and BMI of 14.6 (SD = 1.7) years, and 19.6 (SD = 2.2) kg/m^2^, of which eleven were diagnosed with a restricting subtype and one with the atypical subtype. The demographic characteristics of the all patients are shown in Table 1. No differences were found in any of the variables among the patients who completed the program, those dropped out, and those who declined to participate in the study. No adverse events occurred during the study.

**Table 1:** Demographic characteristics of all patients.

	**Completed program (*n* = 12) *n* (%)**	**Dropped out (*n* = 3) *n* (%)**	**Declined (*n* = 8) *n* (%)**
**Age group**			
11–12	2 (16.7)	1 (33.3)	2 (25)
13–14	6 (50)	1 (33.3)	4 (50)
15–16	3 (25)	1 (33.3)	2 (25)
17	1 (8.3)	–	–
**Anorexia nervosa diagnosis**			
Restricting subtype	11 (91.7)	3 (100)	7 (87.5)
Atypical	1 (8.3)	–	1 (12.5)
**Father's education**			
Primary	2 (16.7)	–	1 (12.5)
Secondary	8 (66.7)	2 (66.7)	6 (75)
University	2 (16.7)	1 (33.3)	1 (12.5)
**Mother's education**			
Secondary	8 (66.7)	2 (66.7)	7 (87.5)
University	4 (33.3)	1 (33.3)	1 (12.5)
**Sibling's number**			
0	1 (8.3)	–	1 (12.5)
1	10 (83.3)	2 (66.7)	5 (62.5)
2	1 (8.3)	1 (33.3)	2 (25)
**Geographical area of residence**			
Urban	8 (66.7)	2 (66.7)	5 (62.5)
Rural	4 (33.3)	1 (33.3)	3 (37.5)

### Psychopathology and Body Image

The differences observed in the psychopathology analyzed by EDI-3 and body image disturbance are shown in Table 2. Regarding psychopathology, small standardized decreases were found in the post-evaluation of bulimia, body dissatisfaction, eating disorder risk composite, low self-esteem, emotional dysregulation, perfectionism and affective problems. In relation to body image, significant, moderately standardized and substantial decreases were observed in the body dissatisfaction (*p* = 0.046), forming nine patients of the total (*n* = 12) with body dissatisfaction in Pre and 5 in Post, and there was a small standardized decrease in the ideal body shape variable.

**Table 2:** Changes in the psychopathology and body image disturbance between Pre and Post.

	**Pre (*n* = 12)**	**Post (*n* = 12)**	**Standardized differences (90% CL)**	**Qualitative assessment**^a^	
**Eating disorder inventory-3**^**b**^					
Drive for thinness	41.4 (7.7)	41.0 (8.0)	−0.06 (0.30)	Unclear	7/72/21
Bulimia	44.4 (2.4)	43.8 (2.3)	–**0.24 (0.42)**	Possibly negative	4/39/57
Body dissatisfaction	41.8 (6.7)	40.6 (8.6)	–**0.23 (0.52)**	Unclear	8/37/54
Eating disorder risk composite	40.6 (6.7)	39.5 (7.8)	–**0.20 (0.40)**	Unclear	5/46/49
Low self-esteem	44.3 (6.4)	42.8 (7.4)	–**0.25 (0.49)**	Unclear	6/36/57
Personal alienation	44.8 (8.3)	45.1 (9.3)	0.02 (0.39)	Unclear	22/62/16
Interpersonal insecurity	44.9 (10.4)	46.0 (10.1)	0.11 (0.32)	Unclear	31/63/6
Interpersonal alienation	47.1 (8.6)	47.0 (8.6)	−0.01 (0.39)	Unclear	18/62/20
Interoceptive deficits	42.3 (8.8)	41.3 (11.2)	−0.17 (0.34)	Possibly trivial	4/52/45
Emotional dysregulation	48.3 (10.1)	45.1 (6.8)	–**0.27 (0.49)**	Unclear	6/35/59
Perfectionism	47.6 (9.5)	45.7 (8.4)	–**0.20 (0.40)**	Unclear	5/45/50
Asceticism	42.4 (6.6)	41.8 (8.6)	−0.13 (0.56)	Unclear	15/43/42
Maturity fears	53.7 (9.8)	53.9 (11.6)	−0.01 (0.18)	Likely trivial	3/93/5
Ineffectiveness	44.3 (7.2)	43.8 (7.8)	−0.08 (0.39)	Unclear	11/59/30
Interpersonal problems	45.7 (9.8)	46.2 (8.9)	0.07 (0.23)	Likely trivial	16/81/3
Affective problems	44.7 (6.8)	42.4 (8.6)	–**0.35 (0.48)**	Possibly negative	3/26/71
Overcontrol	44.2 (7.9)	42.8 (7.3)	−0.18 (0.32)	Possibly trivial	3/52/45
General psychological maladjustment	44.3 (7.7)	43.4 (8.6)	−0.12 (0.31)	Possibly trivial	5/63/33
**Contour drawing rating scale**					
Perceived body shape	5.2 (1.2)	5.2 (0.9)	0.05 (0.46)	Unclear	28/54/17
Ideal body shape	4.6 (1.2)	4.2 (1.0)	–**0.25 (0.36)**	Possibly negative	2/37/60
Body dissatisfaction^c^	0.8 (0.5)	0.4 (0.5)^*^	–**0.69 (0.53)**	**Likely negative**	**1/6/94**

Data are shown as the mean (SD), unless otherwise indicated. Changes in the psychopathology and body image disturbance between Pre and Post were analyzed by the Wilcoxon test.

* p < 0.05. CL, Confidence Level.

a A substantial difference was set at >75%.

b All scales and composite scores of Eating Disorder Inventory-3 are shown in T-scores.

c *The greater values indicate greater body dissatisfaction. Bold indicates a practically worthwhile difference was assumed when the difference score was at least 0.2 of the between subject standard deviation*.

### Quality of Life

Differences in quality of life are shown in Table 3. Significant, large standardized and substantial increases were observed in physical well-being between Pre and Post (*p* = 0.008), the difference exceeding the threshold to consider such clinically relevant changes. There were significant, moderately standardized and substantial decreases in autonomy and parent relation (*p* = 0.021). Small standardized and substantial decreases were found in peers and social support and a small standardized increase in psychological well-being.

**Table 3:** Changes in quality of life between Pre and Post.

	**Pre (*n* = 12)**	**Post (*n* = 12)**	**Standardized differences (90% CL)**	**Qualitative assessment**^**a**^	
**KIDSCREEN-27**					
Physical well-being	42.0 (4.2)	49.0 (8.4)^*^	**1.37 (0.68)**	**Very likely positive**	**99/0/0**
Psychological well-being	45.1 (6.4)	47.5 (10.5)	**0.25 (0.65)**	Unclear	56/33/12
Autonomy and parent relation	49.3 (5.0)	45.8 (3.6)^*^	–**0.60 (0.36)**	**Very likely negative**	**0/3/96**
Peers and social support	51.7 (8.5)	48.7 (11.0)	–**0.41 (0.47)**	**Likely negative**	**2/20/79**
School environment	50.6 (10.2)	51.6 (14.9)	−0.01 (0.43)	Unclear	20/57/23

Data are shown as the mean (SD), unless otherwise indicated. Changes in quality of life between Pre and Post were analyzed by the Wilcoxon test.

* p < 0.05. CL, Confidence Level.

a *A substantial difference was set at >75%. Bold indicates a practically worthwhile difference was assumed when the difference score was at least 0.2 of the between subject standard deviation*.

### Correlations

The correlations of the differences between Pre and Post of all the analyzed variables of the three questionnaires are shown in Table 4. Strong significant negative correlations were observed between body dissatisfaction with physical well-being, psychological well-being with interoceptive deficits, and school environment with emotional dysregulation.

**Table 4:** Correlations of the differences between pre and post.

	**Contour drawing rating scale**			**KIDSCREEN-27**				
	**Perceived body shape**	**Ideal body shape**	**Body dissatisfaction**	**Physical well-being**	**Psychological well-being**	**Autonomy and parent relation**	**Peers and social support**	**School environment**
**Eating Disorder Inventory-3**								
Drive for thinness	0.123	0.189	−0.288	0.486	−0.064	0.011	0.004	0.306
Bulimia	0.000	−0.288	0.213	0.120	−0.306	−0.360	−0.605^*^	−0.170
Body dissatisfaction	0.604^*^	0.028	0.412	−0.081	−0.523	−0.323	−0.205	0.428
EDRC	0.503	0.050	0.260	0.050	−0.512	−0.433	−0.236	0.252
Low self-esteem	0.250	0.107	0.026	−0.421	−0.225	−0.530	0.484	−0.653^*^
Personal alienation	0.090	−0.146	0.437	−0.025	−0.251	−0.418	−0.597^*^	−0.237
Interpersonal insecurity	−0.008	−0.195	−0.077	−0.018	−0.471	0.523	0.208	−0.172
Interpersonal alienation	−0.092	−0.146	0.209	−0.249	−0.084	−0.388	0.050	−0.595^*^
Interoceptive deficits	0.137	−0.416	0.389	−0.163	−0.764^**^	0.039	0.118	0.140
Emotional dysregulation	−0.248	−0.223	0.077	−0.196	−0.295	−0.287	0.123	−0.760^**^
Perfectionism	−0.173	0.390	−0.488	0.270	0.313	−0.239	0.163	−0.204
Asceticism	0.078	−0.625^*^	0.478	−0.058	−0.690^*^	−0.073	−0.263	0.065
Maturity fears	0.069	−0.025	0.105	−0.314	−0.227	−0.439	0.493	−0.519
Ineffectiveness	0.452	0.192	0.266	−0.156	−0.429	−0.672^*^	−0.128	−0.280
Interpersonal problems	−0.023	−0.206	0.103	−0.354	−0.438	0.018	0.304	−0.617^*^
Affective problems	0.060	−0.382	0.206	−0.042	−0.504	−0.246	−0.099	−0.422
Overcontrol	0.265	−0.142	0.052	0.176	−0.452	−0.338	−0.255	0.062
GPM	0.085	−0.176	0.105	−0.068	−0.493	−0.424	0.036	−0.556
**KIDSCREEN-27**								
Physical well-being	0.135	0.483	−0.717^**^					
Psychological well-being	−0.444	0.485	−0.410					
Autonomy and parent relation	−0.406	−0.362	0.051					
Peers and social support	−0.076	0.148	−0.052					
School environment	0.354	0.096	−0.026					

Correlation values are Spearman correlation coefficients. EDRC, Eating Disorder Risk Composite; GPM, General Psychological Maladjustment.

* p < 0.05,

** *p < 0.01*.

### Satisfaction Questionnaire

The descriptive statistics of the satisfaction questionnaire are shown in Table 5, being filled by the 12 patients who completed the program. The average satisfaction was high for each question, reporting at least 75% of patients “agree” or “strongly agree” for each question. The score for the overall rating was 12.8 (SD = 1.9) out of a possible 15 indicating that, on average, these patients showed high levels of satisfaction.

**Table 5:** Descriptive statistics of the satisfaction questionnaire.

	**Strongly disagree (%)**	**Disagree (%)**	**Neither agree nor disagree (%)**	**Agree (%)**	**Strongly agree (%)**	**Mean (SD)**
1. Do you think Pilates has improved your posture (walk better, sit better) and flexibility?	0	0	16.7	41.7	41.7	4.3 (0.8)
2. Do you think Pilates has helped you into feeling better?	0	8.3	16.7	41.7	33.3	4.0 (1.0)
3. Do you think Pilates has made you have less back pain?	0	0	8.3	25.0	66.7	4.6 (0.7)
Overall rating on the Satisfaction Questionnaire	0	2.8	13.9	36.1	47.2	12.8 (1.9)

*Patients who completed the Pilates program (n = 12). Strongly disagree = 1, Disagree = 2, Neither agree nor disagree = 3, Agree = 4, Strongly agree = 5*.

## Discussion

To the best of our knowledge, this is the first study that analyzes the acceptability of a Pilates program evaluating psychopathology, body image and quality of life in children and adolescents with AN. The main findings of this pilot study after the 10-week Pilates program were the significant and substantial increase in physical well-being, and the significant and substantial decrease in body dissatisfaction and autonomy and parent relation.

In recent years, there are few studies that can be found in the scientific literature in which alternative treatments are used in patients with AN. Although we obtained standardized reductions in seven parameters of those that form EDI-3, none of them reached significance. Similar results were found in a study in which the effect of a Yoga program during 8 weeks in young people with EDs was studied, and although the overall scores of the psychopathology decreased, they were not significant after the intervention (42). However, there was a significant decrease in two of the four components of psychopathology that was evaluated in adolescents with EDs after 12 weeks of Yoga (16). The development and evaluation of mental-body activities, in addition to aerobic exercise and massage, for patients with AN seem to be an interesting therapeutic tool that can enhance psychotherapy and contribute to the recovery process, and could even reduce eating pathology (18, 43). The small number of the sample could partially explain the absence of significant reductions in psychopathology. Future studies with a larger number of patients are necessary to analyze whether these reductions could reach significance. Nevertheless, after the results obtained in this pilot study, Pilates could be used as a complementary treatment in children and adolescents with AN since it did not impair any of the psychopathology scores, and seemed to be well tolerated.

The biggest change and statistical consistency after our Pilates program is the decrease in body dissatisfaction. Pilates work with exercises focusing on the sensory increase of internal consciousness, and it has been suggested that this can activate multiple interoceptive channels, such as proprioception from the locomotor- and vestibular system, and also visceroception through controlled breathing (20). These favorable changes can be reflected in the development of body awareness, as previously reported in different studies where Pilates increased body awareness in healthy women (20, 27). Our inference about these results would be in line with the fact that the decrease in body dissatisfaction after the Pilates program in children and adolescents with AN could be related to the increase in body awareness, which is one of the main problems of the illness and is more difficult to treat in these patients. However, body awareness has not been an aim of this study, so future lines of research could deepen this topic.

Regarding the quality of life, an increase in physical well-being was obtained among our patients, in line with other studies where self-perceived general health improved after attending Pilates in the clinical population (24) as in the healthy population (44). Roh (26) studied this relationship and concluded that the improvement of physical self-perception obtained through Pilates in young women strengthens their psychological well-being through their perceived health status. The difference in the physical well-being dimension of the HRQoL after our Pilates intervention exceeds the threshold to determine the minimally important differences (38), indicating a clinically relevant improvement of the physical component of the HRQoL of children and adolescents with AN. The reason for these high increases in the physical well-being of patients may be due to the close relationship between body dissatisfaction and perceived physical well-being. We observed this in our study due to the strong negative association between both variables, the lower the body dissatisfaction, the greater the physical well-being. Although there was only significance in physical well-being in the current research, studies have shown that after a Pilates program, quality of life improves in clinical and healthy populations (22, 25). However, these results should be supported with specific questionnaires that accurately assess the physical well-being comparing Pilates with other types of exercises in this population. We also obtained a strong negative association between psychological well-being with interoceptive deficits and school environment with emotional dysregulation, which seems to be related to the reduced ability to recognize body and inner states in adolescents with AN (45). Therefore, this association in children and adolescents with AN is reflected in the psychological well-being and school environment, by having altered the ability to recognize their own internal states. This may have important clinical implications that would indicate the convenience of including in psychotherapy a greater emphasis on interoceptive sensations and its attention on possible “misleading” internal perceptions, given the subjectivity to interpret them according to the interests of the person. Our patients also showed a decrease in autonomy and parent relation and peers and social support. In a school setting, an increase in psychosocial adjustment was observed in healthy adolescents after the introduction of Pilates in physical education (23), and in children with juvenile idiopathic arthritis, Pilates also had a positive effect on the psychosocial impact of HRQoL (24). These results concerning the family and social environment in our pilot study could be supporting our hypothesis referred to above on the possibility that Pilates favors a greater self awareness (20, 27), since their decrease would indicate a more critical vision and/or less dependent view of the previous context when performing the Pilates program. This could be of great relevance for both the development and recovery of the illness. As in the previous discussion, future studies that prove this inference are necessary.

The clinical impacts that we can observe from this pilot study are that the inclusion of a Pilates program in hospital units for eating disorders in children and adolescents, together with conventional psychotherapy, has a good acceptance by these patients, even finding positive improvements that could help recovery from illness.

### Limitations and Future Directions

There are several limitations to this pilot study. Firstly, as this is a single-arm study, time is the independent variable. The patients had been in psychotherapy for 17 weeks before entering the program and, although it is quite a long treatment time and they continued in it, without having a control group or an active control group, the improvements found may not be unequivocally attributed to the Pilates program. Secondly, the comparatively small sample size is certainly a major limitation of our study, being difficult to enroll a large number of affected children and adolescents for 10 weeks. There are known limitations of all nonprobability samples, including the unknown levels of sampling errors and their lower representativeness. Therefore, future research studying this issue within a control group design and large sample size is clearly necessary to validate and support the current results. Thirdly, although the responses to the satisfaction questionnaire were prepared according to the Likert-scale, the questions could be biased toward positive responses in retrospect. Therefore, open ended questions with several-alternatives would probably be a better way to evaluate the program. Fourthly, objective variables of physical or body changes were not evaluated in this study. Finally, specific questionnaires for associated symptoms such as depression or anxiety were not evaluated in the present pilot study. Thus, future studies should evaluate the depression and anxiety associated with AN after a Pilates program and new lines of research that analyze the possible physical changes that could be found in this population. Furthermore, it would be interesting to observe the possible differences stratified by age in studies with a large sample size. We are aware of the limitations mentioned above and the potentially confounding factors in this pilot study, and therefore the current results must be interpreted with care. However, it deserves to be pointed out that our study was intended as a pilot study and is, to the best of our knowledge, the first to analyze the acceptability of a Pilates program evaluating psychopathology, body image and quality of life in children and adolescents with AN.

After attending the Pilates program three times weekly for 10 consecutive weeks, our patients reported high levels of satisfaction and a high positive overall response. This is certainly a good beginning for future qualitative studies in this field. Therefore, more research is required to prove that Pilates could help in psychopathology and improve the quality of life in this population. We can affirm that through this pilot study it is clearly described how a more definitive study might be conducted, so this represents the first phase of a longer process.

## Conclusion

A Pilates program could help to improve perceived health outcomes by decreasing body dissatisfaction and increasing physical well-being in female children and adolescents with AN, so Pilates seems to be a beneficial complementary treatment in children and adolescents with AN. These findings from our pilot study are encouraging for future research with a substantially larger sample size, representing the first phase of a longer process.

## Data Availability Statement

All datasets generated for this study are included in the article/Supplementary Material.

## Ethics Statement

This pilot study was part of a Clinical Research, which was approved by the Research Ethics Committee of the University Hospital Complex of Huelva (PI 005/16) and registered in www.clinicaltrials.gov (Identifier: NCT03667183). The guidelines of the Declaration of Helsinki, last modified in 2013, were followed and, since all the patients were minors, they and their legal guardians signed written informed consent.

## Author Contributions

SM-S, TM-G, and DM-I: conception and design of the study. SM-S, CM-G, TM-G, and DM-I: acquisition and analysis of data and approved final version. SM-S and CM-G: manuscript draft. All authors contributed to the article and approved the submitted version.

## Conflict of Interest

The authors declare that the research was conducted in the absence of any commercial or financial relationships that could be construed as a potential conflict of interest.
