# Supplementary material for: Psychopathology, Body Image and Quality of Life in Female Children and Adolescents With Anorexia Nervosa: A Pilot Study on the Acceptability of a Pilates Program
Source: Front Psychiatry. 2020 Oct 30;11:503274. doi: 10.3389/fpsyt.2020.503274 (PMC7661430; doi:10.3389/fpsyt.2020.503274)
Supplement: Supplementary file 1 [file Data_Sheet_1.pdf]

Supplementary material: Pilates Mat Program

Warm-up - 10 min:

- Sitting on the mat with legs crossed: lateral head mobilization, inclined head mobilization, neck flexion-extension, neck lateral-flexion, scapular elevation and depression, scapular retraction and protraction with stretched arms, scapular upward and downward rotation with stretched arms.
- Supine with knees bent and feet on the mat: awareness of breathing with the hands the last ribs, pelvis tilt, leg lifts and hip rolls (1).

Pilates exercises (1) – 40 min: The exercises in each class varied depending on each basic Pilates principle. Therefore, there were 6 different classes that were repeated every 2 weeks (since there were 3 classes each week).

- Class 1, Core Control
- Class 2, Axial Elongation
- Class 3, Spinal Articulation
- Class 4, Dissociation of Movement
- Class 5, Limb Alignment and Coordination
- Class 6, Scapular Alignment

Cool-down – 10 min: Child's pose (2) while awareness of breathing and remained quiet. It should be instructed to keep the minds and bodies of the participants as relaxed as possible.

References:

1. Isacowitz R, Clippinger K. Pilates Anatomy. Champaign, IL: Human Kinetics; 2011.
2. Emerson D, Hopper E. Overcoming trauma through yoga: Reclaiming your body. Berkeley, CA: North Atlantic Books; 2011.
